# Inflammatory bowel disease and the psychological difficulties of partners; An interpretative phenomenological analysis

**DOI:** 10.1016/j.qrmh.2026.100052

**Published:** 2026-10-09

**Authors:** Tobias Stark, Anke Erdmann, Claudia Bozzaro

**Affiliations:** a Kiel University, Institute of Experimental Medicine, Medical Ethics, Arnold-Heller-Str. 3, Kiel 24105, Germany; b Kiel University of Applied Sciences, Faculty of Health, Moorblöcken 12, Kiel 24149, Germany; c Kiel University, Institute of Experimental Medicine, Medical Ethics, Arnold-Heller-Str. 3, Kiel 24105, Germany

**Keywords:** Need for help, Depression, Crohn’s disease, Ulcerative colitis, Partner needs

## Abstract

**Introduction:**

Inflammatory bowel disease (IBD) affects not only the patients, but also their partners. This article addresses psychological difficulties for partners and the subsequent need for support.

**Methods:**

Seven partners of IBD patients were, *inter alia*, asked about the effects of the illness on their mental wellbeing in semi-structured qualitative interviews. They were also asked about any potential need for support. The data were analyzed using interpretative phenomenological analysis.

**Results:**

Participants reported psychological difficulties because of the depression that often accompanies IBD. They experienced stress, a sense of helplessness, concerns about the future, and tension between self-care and supporting their partners. From the participants’ point of view, offers of support or help for partners of IBD patients are needed.

**Conclusion:**

This is the first study in Germany to examine the effects of IBD on partners. The results are mainly consistent with those from other international studies dealing with this research question. These have already described most of the psychological difficulties mentioned in this study; however, the tension between self-care and support for the participants’ loved ones suffering from IBD is described in this study for the first time. Medical staff should be sensitive to the effects that IBD has on partners and ask for those effects in order to detect psychological difficulties early and take further steps if necessary. Further offers of support for partners should be considered.

## Highlights

• First study in Germany to examine the effects of IBD on partners.
• Results suppose extraordinary psychological difficulties for partners.
• New findings: Partners’ tension between selfcare and support of the patients.
• Setting up further offers of help for partners should be considered.

## Introduction

Crohn’s disease (CD) and ulcerative colitis (UC) are inflammatory bowel diseases (IBD), which are becoming increasingly prevalent (Ng et al., 2017). The worldwide prevalence of CD is around 84 per 100,000 people and that of UC around 120 per 100,000 people (Heydari et al., 2025). The 12-month prevalence of CD in Germany was 362 per 100,000 people, and that of UC was 421 per 100,000 people in 2018 (Riemekasten et al., 2024). Social support can significantly help to cope with the disease (Lahat et al., 2014, Sewitch et al., 2001). Support from family members seems to be especially important (Lahat et al., 2014). Support from spouses or partners, for instance, is associated with a higher quality of life (QoL) in people suffering from IBD (Katz et al., 2016). Conversely, chronic conditions, generally, and IBD, particularly, affect the family members of people with IBD (Golics et al., 2013).

Literature about the effects of IBD on partners of those suffering from the disease is scarce. Only one qualitative study from 2020 deals specifically with this subject (Thapwong et al., 2022).

There are some other studies dealing with how IBD affects family caregivers, which also include partners. Qualitative interview studies conducted in France (El Hajj et al., 2024) and in China (Liu et al., 2018, Yuan et al., 2025) revealed psychological challenges—e.g., feeling of worry, empathy, impotence and exhaustion—as well as significant impact of the illness on the sexual life of the couple and moderate burden on social activities for caregivers. Psychological challenges as well as negative impacts on relationships, role and responsibilities, day-to-day burden, and suffering for family members were also found in a qualitative study from England (Thapwong et al., 2024) in which only a minority of participants were partners (Thapwong et al., 2024). Further primary studies and especially qualitative studies are needed to find out how IBD affects particularly partners (Loga et al., 2012, Thapwong et al., 2023, Thapwong et al., 2022).

In this study, we defined “partners” as persons who have lived in a steady relationship or marriage with a partner who had been diagnosed with either CD or UC at least six months previously. The relationship had to have lasted for at least six months at the time of the interview, so the patients and their partners had been exposed to the situation for an adequate duration and, thus, be able to report on their experiences and needs associated with their partner’s disease. This article deals with these questions: Which psychological difficulties do partners of IBD patients face, and what are their needs?

## Methods

### Study Design and Recruitment

We conducted qualitative, semi-structured interviews. All procedures were performed in compliance with relevant laws and institutional guidelines. After the study had been approved by the ethics commission of Kiel University (February 17, 2023, reference number D 440/23), recruitment was started via announcements in the outpatients center for inflammatory medicine at a university hospital in Germany. In addition, the project was presented to local self-help groups in the region. Participants were also recruited via contacts and an announcement on the website of the German Crohn and Colitis Association (DCCV).

Purposive sampling was conducted based on the inclusion criteria. Inclusion criteria for the study were having lived in a steady relationship or marriage with a partner who had been diagnosed with either CD or UC at least six months previously. As noted above, this relationship had to have lasted for at least six months at the time of the interview. Participants had to be at least 20 years old. This age was determined because the perceptions of adults who are financially and generally independent of their parents should be focused on in this study. In this group, it is likely that patients’ partners have a greater impact on patients’ coping strategies than their parents. Sufficient knowledge of spoken German or English was necessary in order to make communication possible.

The first contact between researcher and participants took place via either telephone or mail. Participants were mailed a letter of information before the interviews were conducted to inform them about the project. This letter contained information about the background and aims of the project, the contact people responsible and their contact details, and how the interviews were going to be conducted. Participants were informed about the benefits and risks of their participation as well as the voluntary character of the participation. They were informed about the use of their data, data protection, and their right to withdraw their consent at any time.

Four men and three women participated in the study (Table 1).

**Table 1:** Characteristics of Participants.

Gender	Age in years	Disease of the partner	Time since diagnosis in years	Duration of relationship in years
Male	67	UC/CD (diagnosis changed)	26	45
Female	59	UC	19	43
Male	41	UC	1	9
Male	55	CD	23	34
Female	61	Colitis indeterminata (unspecified IBD)	9	10
Male	47	UC	21	2
Female	23	CD	10	5

### Data collection

We collected data in one-time, face-to-face qualitative interviews. The semi-structured interview schedule is based on the example of Thapwong et al. (2022). We asked open-ended questions, regarding the effects of IBD on the participants as well as a potential need for help. For particular effects of IBD, we provided prompts, for instance, for effects on the participants’ relationships. In contrast to Thapwong et al. (2022), we added a question about how much IBD affects the participants, as research on the caregivers of IBD indicated that not every caregiver feels equally burdened (Liu et al., 2018). We also asked if there are any positive effects of IBD on partners. Since the interview schedule of Thapwong et al. (2022) was originally intended for families and not specifically for partners, we changed the wording of the questions to address partners rather than family members in general. The interview schedule was discussed among the research team, and we included additional questions for topics identified as relevant in the literature review.

We conducted a total of seven interviews. Six of them took place at participants’ homes. One interview was conducted in a public café according to the participant’s preference. These locations helped to create the atmosphere of a daily chat and blend the situation of a scientific interrogation into the background (Smith et al., 2021).

No one was present during the interviews that were conducted in the participants’ homes beyond the interviewer and the participant. The interview in the café took place at a table in a secluded part of the café, thus, providing some privacy. The letter of information mentioned previously was discussed with participants directly before conducting the interviews. Subsequently, informed consent to participate in the study, for the interview to be recorded, and for the data to be stored was given by all participants in written form.

The interviews were recorded with a voice recorder and transcribed verbatim. Names of the participants were pseudonymized, and locations or other information which were considered a risk to the anonymity of the participants were paraphrased in the transcripts. Field notes were taken during and after the interviews, whenever something noteworthy occurred. The length of the interviews ranged between 21 and 60 min. The average interview time was 35 min, 48 seconds.

### Data analysis

For our study design, we used interpretative phenomenological analysis (IPA), a method well-suited for examining individuals' personal experiences and how they interpret these experiences (Smith et al., 2021). IPA draws on the foundations of phenomenology and hermeneutics, emphasizing a focus on the individual (Smith et al., 2021). Phenomenology seeks to thoroughly explore human experiences and the significance they hold for people. To achieve this, researchers must "bracket" certain assumptions; in our case, we tried to set aside our perspectives in order to immerse ourselves amidst the experience as subjectively perceived by the participant and reported during interviews.

“Hermeneutics” is a theory of interpretation (Smith et al., 2021) based on the notion that parts are better understood in the context of the whole, and the whole is better comprehended when considering its parts. For this reason, we looked at each statement separately and formulated a core message as stated below. Through the hermeneutic circle, we revisited the data multiple times to develop a comprehensive understanding of it (Smith et al., 2021). The results of the interpretative process were discussed in the research team, and differences were addressed.

Because of the complexity of human phenomena, the analysis in an IPA study should focus on a few cases; a sample size between three and six is considered appropriate (Smith et al., 2021). We analyzed each interview in four steps using the software MAXQDA for coding and Microsoft Excel to note emergent themes and create a printable document to perform our fourth analytical step as explained below.

In the first step, we read and reread the transcripts. We took notes to capture the first impressions.

In the second step, we noted detailed exploratory comments. These comments were subdivided into descriptive comments, linguistic comments, and conceptual comments. The descriptive comments focused on the content of what the participant said, whereas the linguistic comments concentrated on the language used by the participant including pauses, laughter, repetitions, or metaphors. According to interaction studies (Müller et al., 2024), it is impossible not to communicate, so pauses are a source of information which can be interpreted and have to be seen in the context of the specific situation and culture. The other linguistic features were also interpreted under consideration of context and situation. The conceptual comments focused on the participants' perceptions, which they revealed either explicitly or implicitly by what they said.

In the third step, we scanned the data for emergent themes. This process aimed at reducing the volume of detail, while still considering the complexity of participants’ views and feelings. For this reason, we formulated a core message for every statement or remark made by the participant. These core messages served as emergent themes in the context of IPA.

In the fourth step, we clustered the emergent themes and connected them to see which themes recur and how the different themes are connected to each other. We printed a list of all emergent themes, and cut them into pieces, each containing one emergent theme. We placed all emergent themes on a table, arranged and rearranged them regarding similarities, opposites, and other notable features. Methods used within this step were abstraction (finding generic themes for closely related themes), subsumption, polarization (looking for dichotomies), and contextualization*.* At the end of this step, we compiled a table of the structured themes.

Subsequently, we compared the themes of the different cases and sought patterns across the cases regarding the research question. We checked emergent patterns for recurrence across the cases and subsumed them under superordinate themes (Smith et al., 2021).

We present themes in the following text which were mentioned in at least three interviews. This number is seen as the minimum requirement in an IPA study (Smith, 2011).

## Results

Two relevant recurrent themes developed from the analysis. These are the psychological difficulties for partners of IBD patients and the consecutive need for support. The analysis showed that there are different aspects of IBD that result in different scales of psychological difficulties for partners. Specific factors which lead to those psychological difficulties are the depressive comorbidity of IBD patients, stress, helplessness, future concerns, and a tension between self-care and support for their ill partners. Consequently, there is a need for offers of help and support for partners of IBD patients, which most of the participants stated.

### Psychological difficulties for partners

Five of the seven participants reported on psychological difficulties because of their partners’ IBD. When Christian was asked how the experiences in connection with his wife’s IBD influence his mental well-being, he answered directly that it has a negative impact: “Actually negative, let’s say it like that.” Tina was more specific and describes the consequent confrontation with her partner’s illness as a burden: “There were also those times when it was really a constant issue. And that did weigh heavily on me, since it was one of my main concerns.”

Partners perceived these psychological difficulties with different intensity. Sven saw a causal connection to his depression, noting: “This has changed our relationship and me in particular to such an extent that it has actually triggered, at any rate, greatly exacerbated depression.”

Besides the general psychological difficulties caused by IBD, some subcategories were identified. The depressive comorbidity, for instance, that many IBD patients suffer from was a considerable strain on the majority of the partners questioned. In connection with this, Jens spoke about the significantly depressed mood of his partner while suffering from a flare-up.

Then it’s difficult. I know my partner as a really cheerful, energetic, and really lively person. Since the diagnosis there, have been repeated phases where he is tired, listless, exhausted, depending on how severe the flare-up is at the moment.

This quote shows how the disease changed his partner. It seems like his partner became another person since the diagnosis. Jens describes this as “difficult” which refers to the emotional effects of his partner’s transformation.

Christian also reported on the continuously pessimistic and negative attitude of his wife:

Yes, what are further restrictions? She has a pessimistic view of many things now. Everything negatively. Yes, because she’s no longer as m-mobile, is she? We can’t do this any more, we can’t do that any more. Yes. Well, she even more bl-, although, this makes me…. When she’s pessimistic, negative. That…. Yet, sometimes you’re then…. Also annoyed by that. When you get only pessimistic or negative answers.

The anaphora (in the German original an *epiphora*) “We can’t do this anymore. We can’t do that anymore” stresses his wife’s focus on the deficiencies resulting from the disease. “Anaphora” means that words are repeated at the beginning of successive clauses (MacDonald, 2017). "Epiphora" means that words are repeated at the end of successive clauses (MacDonald, 2017). These repetitions show how striking the restrictions due to IBD are for his wife. In the quote, he searches for the right words to describe how her pessimistic attitude affects him and how difficult it is for him to find the right words for this. The repeated sentence breaks indicate that he was probably emotionally affected. In the end, he chose the word “annoyed,” but he did not explain this emotion further. Reading between the lines, it seems as if his partner’s mood affects his own mood in a negative way, maybe even apart from only being annoyed.

Depressive symptoms of the partner suffering from IBD are also mentioned by Norbert. Though the interviewer asked further questions about the effect of the depressive mood of his partner, Norbert did not go further into detail. While speaking about this topic, a certain resistance was perceived by the interviewer:

Norbert: Well, she’s a bit depressed because of that (speaks quieter). Yes.

Interviewer: How does that affect you?

Norbert: Pf, in what way?

Interviewer: Referring to your health or rather mental well-being.

Norbert: Well, yes, a little bit, yes.

The fact that Norbert could not, or perhaps did not want to, go into detail about the effect of his partner’s depressive mood can be interpreted as a hint that this was something bothering him more than he wanted. Another possibility is that there was simply no relevant effect on him. But since he impressed the interviewer as a rather taciturn person who did not want to complain much during the whole interview, the fact that he admitted a negative impact can be appraised as a hint of an emotional strain. The fact that he lowered his voice when speaking about his partner’s depressive symptoms also contributes to this impression.

Another factor impairing the mental well-being of partners is the stress related to the circumstances which accompany the disease. Petra explained, for instance, that the frequent and long bathroom breaks regularly lead to stress in everyday situations:

When there is somehow stress, a doctor’s visit is on the agenda, we are going on holiday, then it immediately kicks in, and I can already, and, as a relative, I know already in advance, oh God, this is going to be stressful again. He’ll be sitting on the toilet for ages again.

Later in the interview, Petra returned to the theme of stress to emphasize that the stress caused by the disease adds to the ordinary stress, which is part of life: “It’s in addition to the stress everyone experiences sometimes, and, well, I would say it causes long-term nervous strain.”

When Norbert was asked how his wife’s disease affects his mental health and well-being, he answered quite briefly that it was sometimes a bit annoying. The seven seconds which he needed for preparing his answer can be interpreted as if he finds it difficult to think of the effects on him. On the other hand, he lowered his voice when speaking about his emotions in this context, which indicates that this topic affects him emotionally. Because of this, it seems more likely that the seven seconds of silence were simply the time he needed to put his emotions into words. As has already been stated above, Norbert impressed the interviewer as rather taciturn, which gives the fact that he mentions this emotional effect even more weight: [7 second silence] “Yes, [quieter] sometimes that was a bit annoying.”

What is more, nearly all of the participants described a feeling of helplessness or powerlessness, which can be an enormous strain on the partners of IBD patients. Referring to the frequent unpredictable bowel urgencies of her partner and the resulting bathroom breaks, the following quote shows how powerless Hanna feels in such situations. With regard to paralinguistic features, she raised her voice. The sentence breaks impressed partly like a stumbling. These paraverbal factors show the psychological strain this powerlessness means to her: “And I can’t. It’s no use to say ‘Come on.’ That’s no use. I have never told him not to make such a big deal out of it, but this is just thi- thi- this isn’t his fault.”

Jens recounted a visit to an amusement park with friends, where his partner had a spontaneous bowel movement in public. Jens felt helpless in this moment, because he could not do more than look for the nearest toilets. The way he stresses the word “catastrophic” demonstrates how much of an effect this experience had on him. He also stressed the word “and” when he stated that it was also a bad experience for him, revealing that this point is important to him. It is obvious that a bowel movement in public is difficult for the one directly concerned. The fact that partners witnessing such an event are also emotionally affected might be less obvious:

Because we were with friends [in an amusement park] in the spring of this year, and it was a *catastrophic* experience for him *and* for me. The weather was bad, people were stressed, and, for him it was difficult to find his way around, to find where the toilets were. And this again led to more stress; this makes the stomach upset, and then, you have to be fast. That was, all in all, a very *bad* experience for both of us, because I, I can’t do more than keeping an eye out.

Helplessness was also mentioned by Tina. She contrasted her sense of helplessness with her compassion for her partner’s situation, first expressing her sympathy and then contrasting it with her sense of helplessness. The second part of the following sentence indicates that she would like to help more than she can, which reflects this feeling of helplessness: “I definitely feel a certain amount of sympathy, but also a bit of helplessness, because I’m not a doctor. And can’t sort of help in any concrete way, at least not in a medical way.”

Future concerns also contribute to the psychological difficulties for partners. Norbert expressed his hope that he and his wife do not have to face another flare-up so that they can spend as much time as possible with their grandchildren. The repeated sentence breaks can also be interpreted as an indicator of the fear of another flare-up: “We hope that the illness stays as it is now, that it doesn’t, doesn’t come back, doesn’t come back again and, like, [2 seconds pause] that we have lots of time left with our grandchildren.”

When asked how the disease might affect the future for her and her partner, Tina stated that she expects IBD to affect the future, since she thinks another flare-up is likely to come:

It’s absolutely concerning that it will, and, to be honest, it’s, well, I think it’s likely also because there have been several flare-ups in the last…. I think it’s already ten years, and we have already tried all the standard medications, so *right now*, it works quite well. But this is currently, I think, the penultimate option of medication, and when this stops working again. So, until now, it was always like this: Medication works for half a year. Then, it stops working. And when it continues like this, we hope that they develop new medication fast enough. But, either way, I think someday, there will be a stressful situation again, which will cause a flare-up. And, I mean, you can’t attach a stoma as often as you want. Some day, that’ll be permanent and so on. And I can imagine that’ll certainly have permanent effects, and that’s not nice, of course. So it’s not my dream.

That she stressed “right now” shows her concerns about a deterioration of her boyfriend’s health. Her experiences with an only temporarily effective medication especially seem to contribute to these concerns. Thus, if patients could rely more on effective medication, one of the partners’ worries could be eased. In the end of the quote, Tina was concerned that her partner might need a permanent stoma, which is something she would prefer to avoid.

Sven was also concerned about another flare-up of his partner’s IBD. As has already been mentioned above, he sees a connection between his depression and his partner’s IBD. In case of another flare-up, he thinks it is likely that he will suffer from another episode of depression:

I know, if there’ll be another flare-up, with all the medication you get, with the cortisone, with the antibiotics, which she can’t tolerate, because the intestinal flora doesn’t work correctly any more, which leads to further problems. Again, this, this I hope that this doesn’t happen. When it happens, it will probably also happen with me again.

This quote raises the question of whether flare-ups in patients are associated with a higher incidence of depression in their partners. Regardless, the quote shows the emotional impact an IBD flare up can have on the patients’ partners.

Furthermore, partners of people with IBD occasionally experience tensions between self-care and supporting their partners. Because of the demands the disease makes on the partners, they struggle to care for their own needs, as the following statement by Hanna shows: “I- I hear, or have often heard, you have to take care of *yourself*, you have to look after yourself. I say to people, how am I supposed to do that?”

Jens reported how he tried to quit smoking. When his partner’s state of health worsened, he wanted to support him and shifted his focus from his smoking cessation program to the well-being of his partner. In the end, his attempt to quit smoking was not successful:

In January, I did a smoking cessation program, hypnotherapy. And it worked wonderfully, for three weeks. And then, [my husband] really got much, much worse again, and I just realized that the pressure of wanting to help him or having to support him somehow, was greater than the desire, to quit smoking. Well, long story short, I started smoking again. I felt better. I could help him.

This is an example of how self-care can compete with the support of the partner suffering from IBD. As stated in the introduction, the partners’ support contributes to the patients’ ability to cope with the disease. It is likely that a mentally healthy partner has more resources to support the patient than a stressed-out partner. Thus, it would be beneficial for partners and patients, if the partners practiced more self-care. Yet, as the quote shows, supporting the patients might stand in contrast to the partners’ self-care.

Sven gave the example of deciding not to go inline skating in order to be with his partner when she suffers from a flare-up. So, he sacrificed his hobbies to support her: “I’m sorry, but I’m not going to do one and a half hours of inline skating when I know she’ll be lying at home. I’d rather be with her.” Sven even went so far as to question whether he should look for help if that means being less able to be with her:

The toughest thing is that any help or support you can get would mean to be, to be basically, somewhere else than with her. And currently, or during the last flare-up, my wish was to be with her as often as possible. So, if I looked for help or support now, in terms of helping with the problem mentally, I would be, I don’t know…. Away for two hours or whatever.

This quote is another example of how the partners’ will to support the patients can stand in contrast to their self-care. We will return to the topic of self-care for partners in the discussion section.

The quotes in this section illustrate in which areas partners of IBD patients face psychological difficulties. The depressive comorbidities of the patients, stress for partners, a feeling of helplessness as well as fears and concerns were mentioned. The tension between self-care and support for the patients is another issue that weighs on partners.

### Partners need offers of help

Psychological stressors result in the partners’ need for help, described by five of the seven participants. Hanna described her wish for help, especially at times when the situation was too much for her: “Well, sometimes it was *really* too much for me, and I wanted to have help in some way.”

Petra complained that there was no clear contact person among the doctors: “What is more, the contact person among the doctors changed constantly, and everyone had to get familiar with the case at first. So, I would have liked to have had one contact, perhaps also for the relatives.” The fact that she wishes to have a contact “maybe also for the relatives” implies that she does not feel like having one at the moment. In this regard, she sees room for improvement in the German healthcare system.

How these offers of help should be organized is seen differently among participants. Most participants would like to have a designated point of contact. Information material for partners was also mentioned. Additionally, self-help groups were proposed. Tina, for instance, talked about getting information regarding how she can help as a partner:

So, basically, some sort of manual, about how I can help. That might reduce this helplessness a bit. For example, referring to diet. Well, I know that I could probably read a lot about which diet is good or not good for Crohn’s disease in terms of nutrition. But there is, for instance, nutritional counseling for those affected, but my boyfriend just doesn’t have the motivation to implement that himself. And maybe I could help more, if I had the specific knowledge in that area or something like that. […] Some point of contact would be good, I guess, just well, it may be a self-help-group or something like that just to talk to someone, […] that you know there is a point of contact as a backup and, that you don’t have to turn to the person affected themselves and make them feel guilty.

Tina hopes that getting more information about the disease might reduce the helplessness she perceives. In particular, she raised the topic of nourishment as she would like to know more about which diet is appropriate for IBD patients in order to support her partner. She added that a point of contact for partners would be helpful and gave the example of a self-help group. The last sentence of the quote is especially interesting, as it shows the partner’s need for an external person to talk to and share their experiences in connection with IBD. Since bowel movements are still a rather shameful topic, it can be difficult to talk about them with other people, though talking to somebody might help with coping.

Sven even suggested a specialized psychotherapeutic point of contact for partners:

Well, I think it’s important to have professional support from a psychologist who understands the clinical picture […] and also knows about what the, the partner is going through in that moment and can provide psychological support so that the partner a) doesn’t fall too deeply into depression and b) can also help the person who is ill.

This quote shows that Sven wants to have someone specialized to talk to about his experiences and emotions while illustrating his fear of relapsing into depression again. The metaphor of “falling” into depression demonstrates a certain sense of helplessness.

All in all, we can see that partners of IBD patients face psychological difficulties. These psychological challenges result in a need for adequate support. The quotations from the interviews show that perceptions of what support services should look like differ.

## Discussion

To the best of our knowledge, this study is the first in Germany to examine the effects of IBD on partners. The results are mainly in line with those of other international studies which deal with this question, indicating that the experiences of partners transcend cultural contexts to a certain extent.

The psychological difficulties for partners have already been described in studies from France (El Hajj et al., 2024), Spain (García-Sanjuán et al., 2019), China (Liu et al., 2018) and the United Kingdom (Thapwong et al., 2024, Thapwong et al., 2023, Thapwong et al., 2022). These studies also partially describe the mediators of these effects such as depressive comorbid symptoms of the patients (García-Sanjuán et al., 2019, Liu et al., 2018), stress (García-Sanjuán et al., 2019, Thapwong et al., 2022), powerlessness (El Hajj et al., 2024, Thapwong et al., 2022), as well as the fears and concerns regarding IBD (García-Sanjuán et al., 2019). The tension between self-care and supporting the ill partners is described here for the first time.

Any discussion about self-care for partners of IBD patients has not yet appeared in the scientific literature to our knowledge. Our study indicates that this might be a considerable aspect of coping with the situation as a partner as it affects various aspects of life. Testimony from the interviews mentions specific aspects of self-care such as the desire to quit smoking or the wish to go inline skating as well as a general feeling of being restricted in one’s self-care. The results of this study and the international studies mentioned show that IBD can be challenging for partners. In this context, self-care can be seen as a way of coping with the stressors related to IBD from a partner perspective. It seems like self-care and coping go hand in hand, so supporting the partners’ self-care efforts might be a key strategy in mitigating the psychological difficulties of partners of IBD patients.

Patients’ other chronic conditions affect the partners of those suffering (Golics et al., 2013); therefore, we searched the literature regarding the self-care of partners connected with other chronic diseases. A study from China investigated the role of self-care in patients suffering from chronic obstructive pulmonary disease and their caregivers who were primarily children and grandchildren of those suffering, but also partners (Cai et al., 2025). Chronic obstructive pulmonary disease is also a chronic condition, but affects the lungs and has different symptoms from IBD. Thus, it might not be very comparable to IBD. Yet, it is a chronic condition and the only one about which we could find any literature investigating the self-care of partners.

According to Cai et al. (2025), self-care can be divided into three components: maintenance, monitoring, and management. A high level of self-care maintenance seems to be associated with an improved QoL for caregivers of patients with chronic obstructive pulmonary disease, whereas a high level of self-care monitoring is associated with a lower QoL (Cai et al., 2025). There were no significant connections found between self-care management and QoL. For a better understanding of the role of self-care management in this context, there is a need for further studies investigating self-care in partners of patients with chronic conditions, especially IBD.

The relatives’ and, thus, the partners’ unmet need for offers of help is also recognized in the international literature (García-Sanjuán et al., 2019, Thapwong et al., 2024, Thapwong et al., 2023, Thapwong et al., 2022). Suggestions regarding what such offers of help could look like are congruent with those evolved in this study. In contrast to Germany (Kucharzik et al., 2024, Sturm et al., 2024), the United Kingdom’s IBD Standards of Care of 2019 recommend offering options for counseling or referral to specialists, not only to patients, but also to their family members if necessary (Thapwong et al., 2024).

Since the problems mentioned in the international studies are mostly the same as those found in this study, it also seems to be important for Germany to consider the needs of partners of IBD patients more. The opportunity to ask the partners how they cope with the situation, especially in primary care, and whether they need help or support should be taken. General practitioners and other healthcare professionals can address partners’ concerns during routine consultations, when appropriate. Depending on the severity of impairment, counseling or psychotherapy could be offered to partners.

Assuming partners of IBD patients were more frequently referred to counseling or psychotherapy specialists, this leads to the question of whether the German healthcare system’s capacity for these interventions could cope with the increase in patients. Currently, most patients seeking psychotherapy wait about four weeks for an initial consultation (Kruse et al., 2024, Singer et al., 2022). Nearly four months pass on average between this initial consultation and the beginning of guideline psychotherapy (Kruse et al., 2024, Singer et al., 2022), indicating that the demand for psychotherapy is much higher than its supply. In order not to overstretch the health system’s capacities, it is important to differentiate between psychiatric disorders and healthy mental reactions to difficult circumstances (Roth & Steins, 2024). Thus, people should only be referred to a psychotherapist if there is an indication based on their symptoms (Roth & Steins, 2024). If there is no indication for psychotherapy, easily accessible alternatives such as coaching or self-help groups could be an alternative. Further studies, especially with a representative sample, should examine how much help and which form of help partners of IBD patients need and which requirements result for the structure of care.

The scientific literature about self-help groups in the context of IBD is scarce. One study revealed that the membership of the Deutsche Morbus Crohn/Colitis ulcerosa Vereinigung (DCCV), the German Crohn and Colitis Association, which also supports self-organized self-help groups, is associated with a significantly higher QoL in IBD patients (Keller et al., 2021). Whether those who declared they were a member of DCCV also took part in meetings of self-help groups was not investigated. Yet, this raises hopes that a DCCV membership or, rather, taking part in a self-help group might also lead to a higher QoL in partners.

We could not find any scientific literature dealing with the topic of self-help groups for partners of IBD patients. According to the DCCV website, their associated self-help groups are also open to partners of those suffering from IBD. When we presented the study in regional self-help groups, we met exclusively patients and were told there was only one date in the year where partners also took part in the meetings. One possible reason could be that patients need an exclusive space where they can talk about their disease-related problems without bothering their partners. Another possible reason could be that the partners’ need for self-help groups is not high enough. Otherwise, a self-help group for partners could have taken place in parallel.

A simple Google search revealed that there is another organization offering help for patients and their relatives. It is called “*CHRONISCH GLÜCKLICH*” which can be translated as “chronically happy,” and operates in Germany. We mailed their northern German group and asked whether their self-help groups were also used by relatives. We also asked which further offers for partners exist and to what extent partners take advantage of these offers. They answered that they had offered occasional meetings with relatives which had many participants. According to CHRONISCH-GLÜCKLICH, the wish for more such offers is mentioned occasionally in their self-help groups for patients. Since the organization is run by people suffering from IBD themselves, offering such meetings on a regular basis goes beyond their capacity at the moment. That is why CHRONISCH-GLÜCKLICH currently focuses on patients and tries now and then to integrate the relatives. However, they try to support partners and relatives individually in acute situations. They do not know about further offers for partners, but they think that there is indeed a demand for such offers.

Along with self-help groups, psychotherapy, and/or counseling, potential alternatives should be considered. Gerlinger et al. (2021) discuss digital health applications as an opportunity to open up new ways of delivering care in Germany. These applications can be generally used in addition to guideline-based psychotherapy and/or pharmacotherapy when a depression is treated with therapy (Gerlinger et al., 2021). There is no intention to use them as an alternative to a guideline-based therapy. Yet, the question whether digital health applications can be used to bridge the waiting time when a depression is diagnosed and the patient considers the waiting time to be inappropriate should be evaluated in further studies.

Another question is if these applications can be used to help people with dysthymia or depressive moods who have not yet been diagnosed with a depression. A digital health application for partners of IBD patients could be an option to help them to deal with the situation and improve their helplessness, independently of the occurrence of psychologic symptoms.

This study has some limitations. Though the small sample size is adequate for an IPA, it seems to be likely that a larger sample size might have reflected more perceptions toward the research question and, thus, resulted in more relevant themes. The regional focus of the recruiting process might also bias the data, since only people from region of Schleswig-Holstein in Northern Germany were recruited.

## Conclusions

• The results of this study give rise to the supposition that partners of people suffering from IBD face extraordinary psychological difficulties.
• The current offers of help for partners in Germany seem to be inadequate. Setting up further offers, such as self-help groups for partners, should be considered.
• Physicians and medical staff should be generally sensitive to these difficulties and their effects on the partners’ mental health in order to detect depression in early stages and prevent any deterioration.

## CRediT authorship contribution statement

**Tobias Stark:** Writing – original draft, Software, Project administration, Methodology, Investigation, Formal analysis, Data curation, Conceptualization. **Anke Erdmann:** Writing – review & editing, Supervision, Software, Project administration, Methodology, Conceptualization. **Claudia Bozzaro:** Writing – review & editing, Supervision, Resources, Project administration.

## Ethics Approval

The study was approved by the ethics commission of Kiel University (February 17, 2023, reference number D 440/23). All procedures were conducted in accordance with relevant institutional and ethical guidelines.

## Declaration of Competing Interests

The authors declare that they have no known competing financial interests or personal relationships that could have appeared to influence the work reported in this paper.

## Data Availability

The original transcripts of the interviews are not available due to data protection.
